# The Anfinsen Dogma: Intriguing Details Sixty-Five Years Later

**DOI:** 10.3390/ijms23147759

**Published:** 2022-07-14

**Authors:** Giorgia Gambardella, Sara Notari, Dario Cavaterra, Federica Iavarone, Massimo Castagnola, Alessio Bocedi, Giorgio Ricci

**Affiliations:** 1 Department of Chemical Sciences and Technologies, University of Rome ‘Tor Vergata’, Via della Ricerca Scientifica 1, 00133 Rome, Italy; giorgia.gambardella@uniroma2.it (G.G.); sara.notari@uniroma2.it (S.N.); cavaterradario@gmail.com (D.C.); bcdlss01@uniroma2.it (A.B.); 2 Dipartimento di Scienze Biotecnologiche di Base, Cliniche Intensivologiche e Perioperatorie, Università Cattolica del Sacro Cuore, 00168 Rome, Italy; federica.iavarone@unicatt.it; 3 Fondazione Policlinico Universitario Agostino Gemelli IRCCS, 00168 Rome, Italy; 4 Laboratorio di Proteomica, Centro Europeo di Ricerca sul Cervello, IRCCS Santa Lucia, 00179 Rome, Italy; maxcastagnola@outlook.it

**Keywords:** ribonuclease, oxidative folding, protein folding, disulfide, protein structure

## Abstract

The pioneering experiments of Anfinsen on the oxidative folding of RNase have been revisited discovering some details, which update the statement of his dogma and shed new light on the leading role of the correct disulfide in the attainment of the native structure. CD analysis, mass spectrometry, fluorescence spectroscopy and enzyme activity indicate that native disulfides drive the formation of the secondary and tertiary structures that cannot be entirely formed in their absence. This opposes a common opinion that these structures are first formed and then stabilized by the native disulfides. Our results also indicate that a spontaneous re-oxidation of a reduced RNase cannot produce a complete recovery of activity, as described by many textbooks; this can be obtained only in the presence of a reshuffling solution such as GSH/GSSG.

## 1. Introduction

The pioneering study of Anfinsen about the denaturation and renaturation of ribonuclease A (RNase) represents one of the most important milestones for the understanding of protein folding, and his conclusions reported in all textbooks of biochemistry are known as the “*Anfinsen dogma*” [1,2,3]. Briefly, he says that at conditions at which folding occurs, the native structure is a unique, stable and kinetically accessible minimum of the free energy. In other terms, the native structure is uniquely determined by the primary structure [4].

As reported by almost all textbooks, two historical experiments made it possible to prove this fundamental property of proteins, i.e., a complete inactive RNase with scrambled disulfides recovers its native activity and the correct disulfides upon incubation with catalytic amount of β-mercaptoethanol (β-ME), which allows a productive reshuffling [5]. A second finding is that a reduced and inactive RNase, obtained in the presence of β-ME and 8 M urea, recovers its full native structure and activity by removing these reagents by dialysis and using atmospheric oxygen as oxidizing agent [1,2,3].

Over the past forty years, my colleagues and I have described these two experiments to our students, exactly as reported above and by almost all textbooks of biochemistry, but when we proposed them to read the original articles, their research gave curious results. Surprisingly, we did not find any papers by Anfinsen describing the removal of urea and β-ME using dialysis, but only by means of gel filtration [6,7]. This appeared to us a no slight difference, since gel filtration causes a very fast separation of urea and β-ME from RNase, while dialysis is a very slow process that makes it possible for a residual amount of the reducing compound to reshuffle incorrect disulfides. More importantly, we noted that the first results obtained after spontaneous re-oxidation of reduced RNase indicated that only 20% of its native activity was recovered starting from a full reduced enzyme [8]. Only a few years later, an almost complete re-activation (80–100%) has been described using particular experimental conditions (i.e., very low protein concentrations, non-physiological temperature, etc.) [9]. Thus, the complete recovery of the native structure seems to require particular conditions and to be a not so univocal and simple process as described in all biochemistry textbooks [1,2,3]. Furthermore, very scarce is the knowledge about the fate of secondary structures during the oxidative folding and during the reductive unfolding of RNase, because one of the most detailed circular dichroism (CD) spectral studies was performed on modified enzyme (carboxymethylated and carboxyamidomethylated) and using not advanced curve-fitting analysis of CD data [10].

The current availability of more accurate and simplified RNase activity methods, fluorescence and mass spectrometry data together with software for the quantitative estimation of secondary structures from CD spectra, encouraged us to reproduce some RNase folding/unfolding experiments to recover additional information.

As it will be clear below, the “*Anfinsen dogma*” [4] will always remain intact, but sixty-five years later a few intriguing details must be refined and corrected, opening surprising conclusions about the role of disulfides in the oxidative folding of this enzyme. We underline that the current work is not a comprehensive reassessment of the “*Anfinsen dogma*”. Rather, it is a study of how experimental conditions dictate the outcome of the refolding of RNase A in vitro. We are also aware that RNase A could exhibit different separable forms enzyme structures able to perform different activities [11], but even these aspects have not been considered in our investigation.

## 2. Results

### 2.1. Does the Reduced and Denatured RNase Spontaneously Recover Its Native Structure and Activity?

In the pioneering report by Sela and co-workers [8], these authors reported that totally reduced RNase obtained using thioglycolic acid as reducing agent and 8 M urea as denaturant, after removal of these reagents and oxidation at pH 7.0 or 8.0 by air bubbling at room temperature (25 °C), no more than 12–19% than the original activity can be restored. A more efficient but not complete recovery of activity was obtained (55% of the native activity) starting from a partially reduced enzyme with one intact native disulfide [8]. In a meticulous study by White [12], eight samples of RNase were reduced using a purified sample of thioglycolic acid. The re-oxidation process gave 85% as mean of reactivation. However, only 50% of the original soluble enzyme was recovered after lyophilization, indicating that relevant amounts of oxidized RNase acquired scarce solubility probably due to incorrect inter- or intra-molecular disulfides. Thus, taking into account this loss, an amount of native and active enzyme of 43% that was formed through this procedure is reasonable. Very similar results were obtained using β-ME as reducing agent [12].

Only a few years later, Anfinsen described an almost complete recovery of activity (80–100%) starting from a full reduced RNase with β-ME in 8 M urea, removal this reagent with a Sephadex G-25 column, and subsequent exposition to air (20 h) at low RNase concentrations (~25 µM) at pH 8.0–8.5 [9].

**Figure 1:**
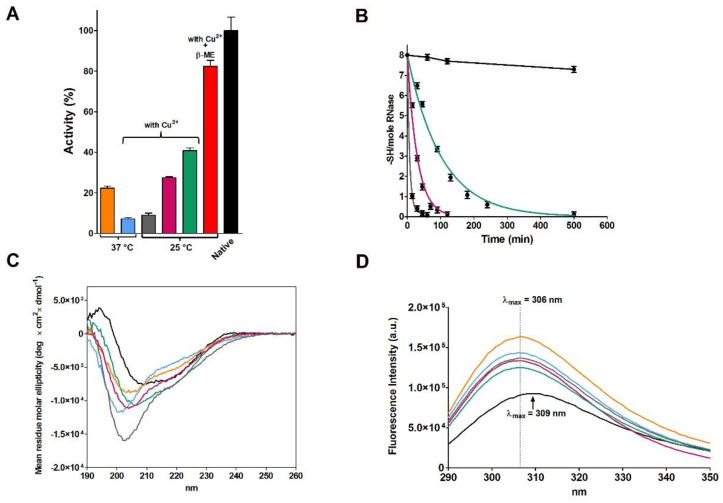
Re-oxidation of rRNase I (14 µM) in 20 mM Tris-HCl pH 8.5 under different conditions. (**A**) Recovery of activity after incubation at 37 °C without (orange column), or in the presence of Cu^2+^ (10 µM) (light blue column). At 25 °C in presence of Cu^2+^ (10 µM) (grey column), Cu^2+^ (1 µM) (pink column), Cu^2+^ (0.3 µM) (green column), and Cu^2+^ (0.3 µM) with β-ME (11 µM) (red column). All the activities are compared to the activity of the native protein (black column). Activities were measured as described under Materials and Methods. The error bars represent the S.D. derived from three independent experiments. (**B**) Time-dependent disappearance of rRNase I sulfhydryl groups in 20 mM Tris-HCl pH 8.5, at 25 °C under different conditions: rRNase I (14 µM) with Cu^2+^ (0.3 µM) (green line), with Cu^2+^ (1 µM) (pink line), with Cu^2+^ (10 µM) (grey line), and with only EDTA (0.5 mM) (black line). The error bars represent the S.D. derived from three independent experiments. (**C**) CD spectra of rRNase I at the end of the re-oxidation process. Native RNase (black line), re-oxidized rRNase I at 37 °C (orange line), re-oxidized rRNase I at 37 °C in the presence of Cu^2+^ (10 µM) (light blue line), re-oxidized rRNase I at 25 °C in the presence of Cu^2+^ (0.3 µM) (green line), Cu^2+^ (1 µM) (pink line), and Cu^2+^ (10 µM) (grey line). All CD spectra were performed with a protein concentration of 1.25 µM in 10 mM sodium phosphate buffer pH 7.4, at 25 °C. (**D**) Intrinsic fluorescence emission spectra of native RNase and rRNase I at the end of re-oxidation process. Native RNase (black line), re-oxidized rRNase I at 37 °C (orange line), re-oxidized rRNase I at 37 °C with Cu^2+^ (10 µM) (light blue line), re-oxidized rRNase I at 25 °C with Cu^2+^ (0.3 µM) (green line), Cu^2+^ (1 µM) (pink line), Cu^2+^ (10 µM) (grey line). All the fluorescence emission spectra were recorded with a protein concentration of 1.25 µM in 10 mM sodium phosphate buffer pH 7.4, 25 °C.

Our attempts to reproduce these results were disappointing in both kinetics of disulfide formation and activity recovery under various temperature and protein concentrations. More precisely, starting from the reduced and denatured RNase I (rRNase I and other rRNase samples, i.e., rRNase II, III and IV, differ mainly in the chromatographic purification step, see Materials and Methods), incubation at pH 8.5 with very low protein concentration (14 µM) did not give a complete oxidation before two days had elapsed (Table 1), and no more than 20–30% of the original activity (Figure 1A and Table 1). We believe that the faster oxidation observed by Anfinsen and Haber (20 h) [9] was possibly due to an amount of spurious metals, a not uncommon flaw in buffer preparations in the mid-20th century and, in traces, even present in our experiments (Figure 1A). In fact, EDTA strongly inhibits the re-oxidation and the presence of trace of Cu^2+^ and other metals greatly enhances the process as also observed by other authors (Figure 1B) [10,13]. The presence of 10 µM Cu^2+^ gave very low recovery of activity compared to the 0.3 µM Cu^2+^ possibly due to a fast and tumultuous disulfide formation (mostly incorrect).

For sake of simplicity in the present work, we will continue to use the term auto-oxidation or spontaneous oxidation (used by Anfinsen and many other authors and textbooks) instead of the more correct “metal catalyzed oxidation”.

The CD spectrum of the partially active and fully re-oxidized rRNase I at pH 8.5 with or without Cu^2+^ did not overlap the one of the native enzyme, indicating lower amounts of beta sheets and turns (Figure 1C and Table 2). The intrinsic fluorescence of the six tyrosines of rRNase I gave further insights. In fact, the spectrum of the re-oxidized protein does not overlap the one of the native enzyme, despite all disulfides were formed. As it will be discussed below, this is a signal that tyrosines, in the re-oxidized rRNase I, reside in an increased non-polar environment compared to the native enzyme (Figure 1D) [14]. This indicates different tertiary structures formed as a consequence of non-native disulfides. Interestingly, the presence of sub-stoichiometric amount of β-ME (11 μM against 14 μM of rRNase I) allows a relevant recovery of activity (82%) in the range of that found by Anfinsen (80–100%) (Figure 1A and Table 1).

**Figure 2:**
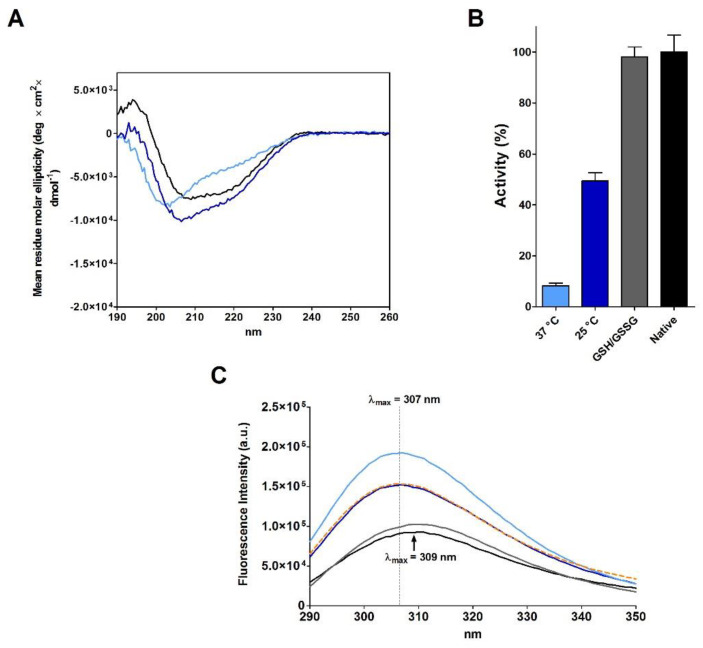
Analyses at different stages of rRNase II (14 µM) during re-oxidation process in 20 mM Tris-HCl pH 8.5 under different conditions. (**A**) CD spectra of rRNase II at the end of the re-oxidation process. Native RNase (black line), re-oxidized rRNase II at 37 °C (light blue line), and re-oxidized rRNase II at 25 °C (navy blue line). All CD spectra were performed with a protein concentration of 1.25 µM in 10 mM sodium phosphate buffer pH 7.4, at 25 °C. (**B**) Recovery of activity of re-oxidized rRNase II at 37 °C (light blue column), at 25 °C (navy blue column), and residual activity of re-oxidized rRNase II 2.0 µM at 30 °C in 50 mM sodium phosphate buffer pH 7.5 with GSH/GSSG (2 mM/0.4 mM) and 5 mM EDTA (grey column). All the activities are compared to the activity of the native protein (black column). Activities were measured as described under Materials and Methods. The error bars represent the S.D. derived from three independent experiments. (**C**) Intrinsic fluorescence emission spectra of native RNase (black line), rRNase II (orange dotted line), re-oxidized rRNase II at 37 °C (light blue line), re-oxidized rRNase II at 25 °C (navy blue line), and re-oxidized rRNase II at 30 °C in 50 mM sodium phosphate buffer pH 7.5 with GSH/GSSG (2 mM/0.4 mM) and 5 mM EDTA (grey line). All the fluorescence emission spectra were recorded with a protein concentration of 1.25 µM in 10 mM sodium phosphate buffer pH 7.4, 25 °C.

As Anfinsen, after the reduction step, removed β-ME and urea using a Sephadex G-25 column equilibrated with acetic acid (pH 3.5), we also replicated this procedure obtaining a new reduced RNase (rRNase II, see Materials and Methods). The full oxidation of this reduced protein is again slow (4.5 day at 25 °C) (Table 1) and CD analyses suggest a secondary structure only approaching that found in the native enzyme, showing a lower amount of beta structures (Figure 2A and Table 3). The complete oxidized enzyme also displays 49% of the native RNase activity, which represents the maximum recovery reached in all our experiments without a classical reshuffling mixture (GSH/GSSG) or without a sub-stoichiometric amount of β-ME (Figure 2B and Table 1). The intrinsic fluorescence spectra also indicate that oxidized protein acquired a tertiary structure different compared to the native RNase with the tyrosines inserted in a more hydrophobic environment (Figure 2C). This represents a further indication that incorrect disulfides oppose a complete re-formation of native secondary and tertiary structures of this enzyme. 

Furthermore, the replica of a famous experiment of re-oxidation of rRNase II at extreme low concentrations (1.8 µM), capable of recovering 100% of the original activity in 1 h, as described by Anfinsen [7], gave disappointing results. The full re-oxidation of rRNase II requires three days (Table 1), which must be compared to one hour described by Anfinsen [7]. Moreover, the recovery of activity is only of 23% (Table 1).

The results claimed by Anfinsen for a spontaneous complete recovery of activity generated the belief that the reduced RNase spontaneously forms all its native structure and that proper disulfides can be formed later as stabilizers. This is what is described in many textbooks [1,2,3]. However, this is disputed by evidence that insufficient recovery of the original structure and activity can be obtained, likely due to the formation of incorrect disulfides. In fact, more recently, a quasi-stochastic mechanism for its oxidative folding has been proposed [15,16]. In addition, more recent studies agree with our results, observing that incomplete native structure can be recovered using a particular oxidizing agent [17]. Actually, we obtained a full recovery of the native activity and an emission spectrum close to the native one only after incubation with a reshuffling solution of GSH/GSSG (Figure 2B,C). This agrees with later studies which observed that the use of a mixture of reduced/oxidized DTT may contribute to restore the native conformation [18,19]. As above reported, also traces of β-ME (Table 1) can produce very high recovery of the original activity.

### 2.2. Reduction of RNase under Different Conditions

**Figure 3:**
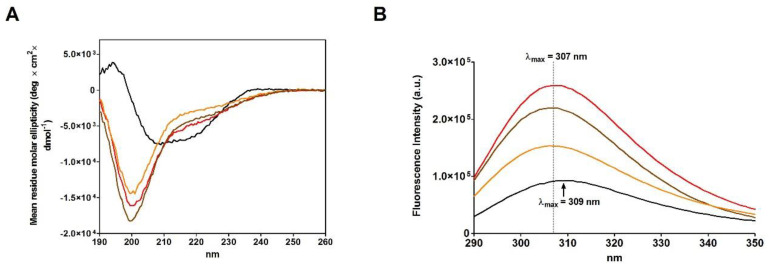
Spectroscopic analysis of the three rRNase I, rRNase II and rRNase III. (**A**) CD spectra of native RNase (black line), rRNase I (red line), rRNase II (orange line), and rRNase III (brown line). (**B**) Intrinsic fluorescence emission spectra of native RNase (black line), rRNase I (red line), rRNase II (orange line), and rRNase III (brown line). The CD spectra and the fluorescence emission spectra were recorded with a protein concentration of 1.25 µM in 10 mM sodium phosphate buffer pH 7.4 at 25 °C.

The recovery of activity, obtained using different reduction procedures, prompted us to investigate if different reductive conditions may generate structurally different rRNases (see Materials and Methods). CD spectra suggest that rRNase I and rRNase II display not negligible differences in secondary structure possibly responsible for the observed different restoration of activity (Figure 3A). For example, rRNase I shows a lower amount of beta structures compared to the rRNase II (Table 4).

The rRNase I displays a much more fluorescence emission compared to the rRNase II confirming also different tertiary structures (Figure 3B). 

### 2.3. RNase Reduction in the Absence of Denaturing Agent

Anfinsen used 8 M urea and a relevant amount of β-ME (about 0.6 M) to reduce all the four disulfides in RNase [9]. The presence of urea was necessary because, in its absence, only a partially reduced RNase can be obtained, even after long incubation times at pH 8.5 [12]. In this way, however, nobody could distinguish between the effect due to urea and that due to the disulfide breakdown on activity and structure. The use of a stronger reducing agent such as DTT allows us to explore what happens to the enzyme activity and structure when the disulfides are progressively broken in the absence of a denaturing solution. DTT is a reagent of particular interest as its oxidized form is greatly stabilized being a cyclic disulfide, and it cannot form mixed disulfides when it interacts with protein cysteines. 

**Figure 4:**
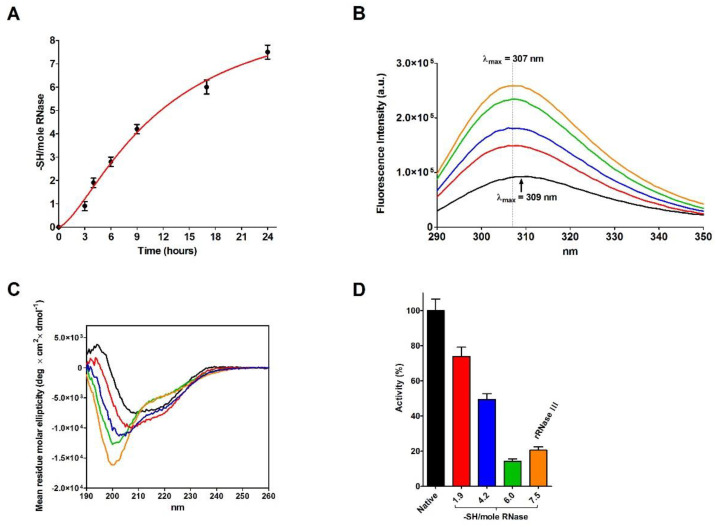
Analyses at different stages of RNase reduction. (**A**) Time-dependent formation of -SH per mole of rRNase III under reducing conditions (see Materials and Methods). The error bars represent the S.D. derived from three independent experiments. (**B**) Intrinsic fluorescence emission spectra of native RNase (black line), rRNase III (orange line), rRNase III with 6.0 -SH/mole (green line), 4.2 -SH/mole (blue line), and 1.9 -SH/mole (red line). All the fluorescence emission spectra were recorded with a protein concentration of 1.25 µM in 10 mM sodium phosphate buffer pH 7.4, 25 °C. (**C**) CD spectra of native RNase (black line), rRNase III (orange line), rRNase III with 6.0 -SH/mole (green line), 4.2 -SH/mole (blue line), and 1.9 -SH/mole (red line). All the CD spectra were recorded with a protein concentration of 1.25 µM in 10 mM sodium phosphate buffer pH 7.4, 25 °C. (**D**) Residual activity of rRNase during reduction pathway: native RNase (black column), rRNase III with 1.9 -SH/mole (red column), 4.2 -SH/mole (blue column), 6.0 -SH/mole (green column), and rRNase III (7.5 -SH/mole, orange column). All the experiments were performed as described under Materials and Methods. The error bars represent the S.D. derived from three independent experiments.

The incubation of native RNase with DTT causes a progressive and complete reduction of the four disulfides even in the absence of urea (Figure 4A). During this process, a relevant increase in the emission fluorescence of tyrosines has been found probably due to a shift in these residues toward a more hydrophobic environment and then to a change in the tertiary structure (Figure 4B) [20].

A comparison of the CD spectra of the rRNase I and II, obtained in the presence of urea, with rRNase III, obtained in absence of urea (see Materials and Methods), suggests very similar reduced structures showing a relevant lack of secondary structures more evident for beta-sheets compared to the native form (Figure 3A and Table 4). Therefore, the presence of urea is not essential for the modification of the secondary structure, only determined by breakage of disulfides (Figure 3A and Figure 4C).

Surprisingly, the reduction of only one disulfide is able to generate some not negligible structural changes by lowering the amount of beta structures and reducing the activity to 74% (Figure 4C,D and Table 5). Other prominent structural and activity perturbations occur when other disulfides are broken step by step (Figure 4C,D and Table 5).

**Figure 5:**
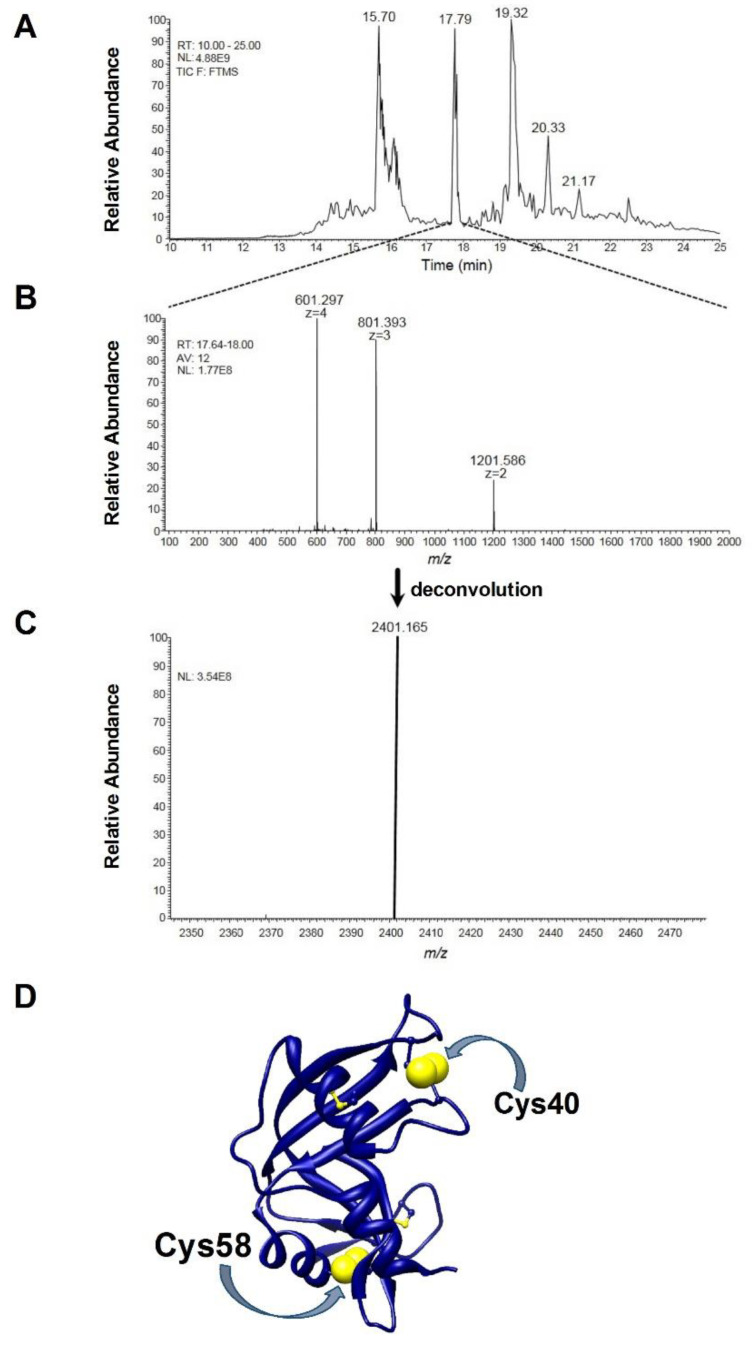
MS and MS/MS analysis. (**A**) nano-HPLC-ESI-MS profile of the tryptic digest of the partially reduced rRNase III with 1.9 −SH/mole (1.25 μM). TIC: total ion current; NL: normalization level. (**B**) ESI spectrum of the chromatographic peak eluting in the 17.64–18.00 min retention time (RT) interval, resulting from the average (AV) of 12 ESI spectra. (**C**) Deconvolution of the ESI spectra reported in panel (**B**). The experimental [M + H]^+1^ monoisot. = 2401.165 *m/z* is very close to the theoretical [M + H]^+1^ monoisot. = 2401.164 *m/z* of the tryptic fragment 40–61 of RNase (CKPVNTFVHESLADVQAVCSQK) with a non-native disulfide bridge between Cys40-Cys58. The poor MS/MS spectra deriving from the HCD fragmentation of this connected peptide did not allow the confirmation of its structure, neither the other poor HCD MS/MS spectra permitted the characterization of the structure of other not-identified tryptic fragments deriving from a highly connected molten globule eluting in the initial and final part of the nano-HPLC-ESI-MS profile of panel (**A**) at different reduction ratios. (**D**) Three-dimensional structure of native RNase from bovine pancreas is represented in blue ribbons; cysteines are in ball and stick, the Cys40 and Cys58 are displayed in blue and yellow spheres.

A further indication that the breaking of just one disulfide is responsible for important structural changes comes from mass spectrometry data. When the reduction process with DTT was stopped when only one disulfide was broken, the partially reduced protein was incubated with bromopyruvate, a reagent that alkylates reduced cysteines in a few seconds. Digestion and ESI mass analysis of this modified protein revealed a quantitatively relevant amount of one peptide with an experimental [M + H]^+1^ monisot. = 2401.166 *m/z*, corresponding to the fragment 40–61 of RNase (CKPVNTFVHESLADVQAVCSQK) with a non-native disulfide bridge, i.e., Cys40-Cys58 (theoretical [M + H]^+1^ monisot. = 2401.164 *m/z*) (Figure 5A–C). Both these cysteines are very distant in the native protein and linked with Cys95 and Cys110, respectively (Figure 5D).

This finding is a strong indication that one of the two free cysteines, originated by DTT reduction of a single disulfide, interchanges with another distant disulfide and this becomes possible only if relevant secondary and tertiary structural changes occurred.

Only a few experimental studies about the partial reduction of RNase have been performed, but they support our results. In particular, the study of Li and co-workers [21] identified the Cys40-Cys95 as one of the two disulfides first broken upon selective reduction. Even the theoretical study of Krupa and co-workers identified Cys40-Cys95 as the more susceptible disulfide to be the first cleaved [22].

### 2.4. Refolding of rRNase Avoiding Disulfide Formation 

**Figure 6:**
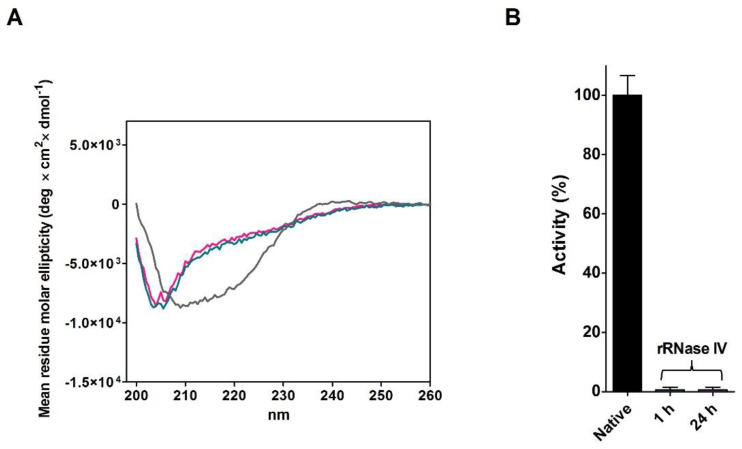
Analyses of rRNase IV incubated under reducing conditions at 37 °C. (**A**) CD spectra of native RNase incubated in urea for 1 h without DTT (grey line), rRNase IV incubated in 0.05 M urea, 0.2 mM DTT and 1 mM EDTA for 1 h (emerald line), and rRNase IV incubated in 0.05 M urea, 0.2 mM DTT and 1 mM EDTA for 24 h (pink line). The CD spectra were recorded with a protein concentration of 1.25 µM in 10 mM sodium phosphate buffer pH 7.4 at 25 °C (see Materials and Methods). (**B**) Activity of rRNase IV incubated as in panel (**A**) and activity of native RNase (black column). All the experiments were performed as described under Materials and Methods. The error bars represent the S.D. derived from three independent experiments.

A further experiment, never done in the past, was designed to observe the evolution of the structure and activity of the reduced RNase, when incubated avoiding the disulfide formation (rRNase IV, see Materials and Methods). The rRNase IV was incubated at pH 7.4 in the presence of 0.2 mM DTT. Either after one hour or after 24 h, no appreciable recovery of activity was observed. CD spectra also indicate a relevant absence of alpha structures (~−70%), and an evident but not dramatic lack of beta structures (~−30%) (Figure 6A,B and Table 6).

We believe this result to be important as, for the first time, it suggests that without disulfides a reduced RNase is completely unable to re-form native-like secondary structures.

### 2.5. The Effect of Urea on the Activity of the Native RNase

In 1955, Anfinsen described a curious behavior of RNase. Apparently, prolonged incubation of this enzyme in 8 M urea at 5 °C did not produce any loss of activity [23]. This result was not disproved or modified later but only corrected in its interpretation. In fact, in 1989, Anfinsen, commenting his own work, says that it represents “*a beautiful example of how an entirely acceptable conclusion can be reached that is entirely wrong because of the paucity of knowledge at that particular time*” [24] (pp. 197–198). This refers to his previous (wrong) conclusion that an ordered shape of a protein is not strictly needed for its catalytic function if the structure of the active site is intact. In fact, further studies revealed that some apparent increment of activity occurs at high urea concentration probably due to an increased solubility of the reaction product or to a denaturation of the RNA thereby making it more available to the digestion by the enzyme [25]. In reality, the RNase activity is slightly lowered at similar urea concentration [25].

**Figure 7:**
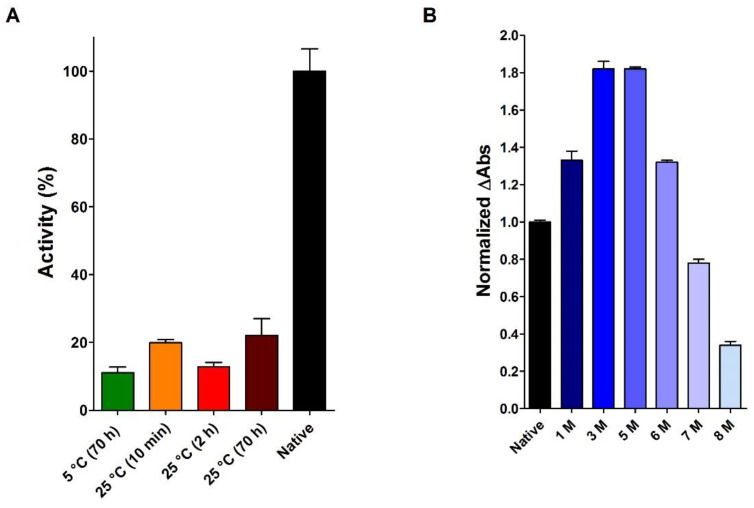
Activity of native RNase (2 µM) with different urea concentrations. (**A**) Residual activity of native RNase incubated for different times in 8 M urea at different temperatures: 70 h at 5 °C (green column), 10 min. at 25 °C (orange column), 2 h at 25 °C (red column), and 70 h at 25 °C (brown column). The activity of the native protein is also reported (black column). The experiments were performed as described under Materials and Methods. (**B**) Normalized activity of native RNase incubated for 1 h at 25 °C under different urea concentrations from 1 to 8 M urea (six shades of blue columns). All the activities are compared to the activity of the native protein (black column). The experiments were performed as described under Materials and Methods. The error bars represent the S.D. derived from three independent experiments.

Thus, the full preservation of the RNase activity in 8 M urea, as reported by Anfinsen, is certainly a curious result, and my co-workers and I have been interested in replicating this experiment. However, as shown in Figure 7A the presence of 8 M urea strongly inhibits RNase either after short or long time of incubation. Our experiments also confirmed an apparent increase in activity at lower urea concentrations (up to 6 M) and a slight decrease in activity at 7 M urea (Figure 7B). Thus, the lack of inhibition due to 8 M urea found by Anfinsen and co-workers [23] was possibly due to some overestimation of the urea concentration occurred in their activity measurements.

## 3. Discussion

Although during the past sixty-five years a lot of studies have been published about the oxidative folding and reductive unfolding of RNase, data present in this paper allow us to update some details not previously underlined.

First of all, the always-cited Anfinsen’s experiment of a spontaneous complete activity recovery of RNase by means of a simple exposition of the reduced enzyme to air oxygen [4,26] cannot be easily reproduced: no more than 30–50% of the original activity was obtained under variable conditions of pH, enzyme concentrations and reduced RNase preparations. Thus, the correct folding does not appear so straight and simple process. The presence of a reshuffling mixture is necessary to reach around 100% of a native activity (Figure 2B), as also noted by other researchers [13,20,27]. We speculate that the complete restoration of activity, as observed by Anfinsen, was probably due to traces of residual β-ME in the reduced enzyme sample. Such possibility is not unlikely seeing the not complete separation of reduced RNase from β-ME in the G-25 column, reported in Figure 1 in the study by Anfinsen and Haber [9]. If present, this contamination may promote a reshuffling of the uncorrected disulfides. The almost complete recovery of activity in the presence of traces of β-ME as obtained by us and shown in Figure 1A is a good support for this hypothesis. Alternatively, some activity overestimation could be another factor due to the complexity of the enzymatic assay used in the past [28]. Incidentally, the statement reported in many textbooks that Anfinsen removed denaturing and reducing agents by means of dialysis has no confirmation in the literature [4,6,7,8,9,29,30]. 

Data concerning the secondary structure, obtained by CD analysis during the spontaneous oxidation pathway, allow us to obtain important structural information not acquired in the past on the un-modified RNase. In parallel to the observed partial recovery of native activity, also the CD spectrum differs significantly from that of the native enzyme. More in detail, when all disulfide are reformed, a lack of beta structures is evident in the totally re-oxidized rRNase I and II (Table 2 and Table 3).

Changes occurring in the secondary structures during the reductive pathway in the absence of urea fulfilled other important information. All structural and catalytic properties of RNase reduced under mild conditions of temperature and pH (rRNase III) indicate that this enzyme displays a different secondary structure with a lower amount of beta structures and a progressive loss of activity (Figure 4C,D and Table 5). In particular, the simple reduction of a single disulfide is able to trigger relevant structural changes as suggested by the formation of the non-native Cys40-Cys58 disulfide, both residues very distant in the native structure (Figure 5A–D). Similarly, incubating the rRNase IV at physiological pH and temperature values under constant reducing conditions, the enzyme does not recover its original secondary structures and shows also a negligible enzyme activity (Figure 6A,B and Table 6). Therefore, the reduced enzyme is unable to fold back into a native conformation except by allowing it to reform its natural disulfides.

In conclusion, the well accepted assumption that RNase refolds spontaneously into correct secondary and tertiary structures and that disulfides consolidate such natural architecture must be completely refused. An opposite scenario it seems more reasonable, i.e., that the native disulfide formation is the necessary requirement to assemble the polypeptide into complete and correct alpha and beta structures. Refined CD analyses, not disposable sixty years ago, advise for this conclusion. 

Obviously, CD spectra do not fulfill any information on the tertiary structure, but some indications can be obtained by the changes of the fluorescence emission of the six tyrosines present in RNase. For example, the relevant increase in fluorescence emission at 300 nm (compared to the one of the native enzyme) observed in all the re-oxidized RNases under different conditions, points for different tertiary structures beside the above described different secondary structure. More precisely, it appears that in these non-native structures, tyrosines are internalized in a more hydrophobic environment compared to the native enzyme [20]. Alternatively, a greater distance of these tyrosines from the disulfides, known as fluorescence quenchers, could explain these results, but again, this would confirm tertiary structure modifications [14]. In fact, the increase in fluorescence observed in all reduced enzyme forms cannot be simply due to the cleavage of disulfide because a relevant increase in fluorescence is observed when uncorrected disulfides are formed (Figure 2C).

The reshuffling procedure, necessary for a complete recovery of activity indicates that some uncorrected disulfides are first formed with only partial development of secondary structures. 

**Figure 8:**
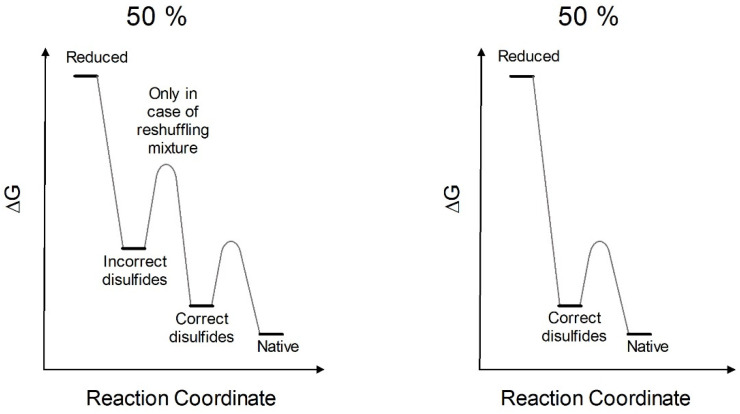
Putative energetic profile of RNase oxidative refolding pathways. From the totally reduced protein to native conformations through kinetic traps (incorrect disulfides) with reshuffling mixture (e.g., GSH/GSSG); without reshuffling mixture, it will not be possible to reach native conformations (left panel). From totally reduced protein to native conformations through intermediates (correct disulfides) (right panel). These schemes derived from the best experimental conditions (described in this study) of reduction and re-oxidation (i.e., rRNase II). The percentage of 50% is referred to the productive conformations that evolved to the native state and is derived from the enzymatic activities as reported in Figure 2B.

The continuous breaking and reformation of these disulfides, promoted by catalytic amount of reducing agent, or by a mixture of GSH/GSSG, is the mechanism needed for the formation of all correct disulfides. This event is crucial and precedes the definitive folding into all secondary and correct tertiary structures [15]. The thermodynamic profile toward the native structure can be now schematized, where, in the most favorable condition, only about 50% of RNase recovers spontaneously its native conformation and correct disulfides, while a second 50% falls into an un-proper energy hole characterized by incomplete secondary structure, improper disulfide and very low activity (Figure 8). A reshuffling procedure is necessary to convert it into the native conformation with natural disulfides. Assuming correct this mechanism, only four steps of reshuffling are needed to reach 94% of the native activity. This occurs only using rRNase II as lower levels of native conformations (20–30%) are obtained with rRNase I or rRNase III.

In conclusion, this study describes new details of the in vitro oxidative pathway described many years ago by Anfinsen, but we are aware that the in vivo process can proceed in different ways. The recent discovery of an ultra-rapid glutathionylation of Cys95 [31,32] when reduced RNase is in the state of molten globule and similar phenomenon found in other proteins (i.e., serum albumin, lysozyme, chymotrypsinogen and trypsinogen) [33,34,35,36] are an intriguing indication in that direction.

## 4. Materials and Methods

### 4.1. Chemicals and Reagents

Ribonuclease A (RNase) from bovine pancreas (Type XII-A, 75–125 Kunitz units/mg protein), bromopyruvic acid, copper sulfate, dithiothreitol (DTT), ethylendiamminotetreaacetic acid (EDTA), 5,5′-dithiobis(2-nitrobenzoic acid) (DTNB), L-glutathione (GSH), oxidized glutathione (GSSG), β-mercaptoethanol (β-ME), ribonuclease A activity kit and all other reagents were purchased from Sigma-Aldrich (St. Louis, MO, USA). 

The GSH solutions were prepared immediately before use and the GSSG concentration was less than 1% as assayed by standard analytical procedures.

All organic solvents were of LC-MS grade. Acetonitrile (ACN), methanol (MeOH), formic acid (FA) and water were from Merck (Darmstadt, Germany). Trypsin (Gold MS Grade) was from Promega (Madison, WI, USA).

### 4.2. Protein Reduction

RNase concentration was evaluated by an extinction coefficient of 9440 M^−1^ cm^−1^ at 280 nm [37].

RNase reduction was carried out with a protein concentration of 0.13 mM in 10 mM sodium phosphate buffer pH 7.4, 8 M urea, 1 mM EDTA and 10 mM DTT at 37 °C for 120 min. The reduced protein was passed through Sephadex G-25 column (1 × 20 cm) equilibrated in 10 mM sodium phosphate buffer pH 7.4 at 25 °C (through the manuscript the reduced protein after this process is indicated as rRNase I), or G-25 equilibrated in 0.1 M acetic acid at 25 °C (through the manuscript the reduced protein after this process is indicated as rRNase II). The number of -SH/mole of the eluted protein was titrated with DTNB at pH 8.0 (ε_M_ TNBS^−^ = 14,100 M^−1^ cm^−1^), 25 °C [38].

Alternatively, reduction of RNase 0.1 mM (or 0.2 mM), in absence of denaturant, was carried out in 0.1 M sodium phosphate buffer pH 7.4, 1 mM EDTA and 100 mM DTT at 37 °C for different times. After reduction, the excess of DTT was removed by passing through a Sephadex G-25 column (1 × 20 cm) equilibrated with 10 mM sodium phosphate buffer pH 7.4 at 25 °C (through the manuscript, the reduced protein after this process is indicated as rRNase III). The number of -SH/mole of the eluted protein was titrated with DTNB at pH 8.0 (ε_M_ TNBS^−^ = 14,100 M^−1^ cm^−1^), 25 °C. 

The preparation of rRNase IV for the characterization of conformational changes in the presence of the reducing agent DTT was performed as above described (starting from a protein concentration of 0.3 mM) for the preparation of rRNase I omitting the Sephadex G-25 chromatography. The rRNase IV was diluted to a final concentration of 2 µM in 10 mM sodium phosphate buffer pH 7.4, 1 mM EDTA, 0.2 mM DTT and 0.05 M urea. The solution was incubated at 37 °C for 1 and 24 h before the CD analyses and enzyme activity assays. 

### 4.3. Re-Oxidation of rRNase

The re-oxidation of rRNase I, rRNase II and rRNase III was carried out in sixteen different manners: (i). rRNase I (14 µM) in 20 mM Tris-HCl buffer pH 8.5 at 37 °C; (ii). rRNase I (14 µM) in 20 mM Tris-HCl buffer pH 8.5 at 25 °C; (iii). rRNase I (9.2 µM) in 20 mM Tris-HCl buffer pH 8.5, Cu^2+^ 10 µM at 37 °C; (iv). rRNase I (14 µM) in 20 mM Tris-HCl buffer pH 8.5, Cu^2+^ 0.3 µM at 25 °C; (v). rRNase I (14 µM) in 20 mM Tris-HCl buffer pH 8.5, 11 µM β-ME, and Cu^2+^ 0.3 µM at 25 °C; (vi). rRNase I (14 µM) in 20 mM Tris-HCl buffer pH 8.5, Cu^2+^ 1 µM at 25 °C; (vii). rRNase I (14 µM) in 20 mM Tris-HCl buffer pH 8.5, Cu^2+^ 10 µM at 25 °C; (viii). rRNase II (14 µM) in 20 mM Tris-HCl buffer pH 8.5 at 37 °C; (ix). rRNase II (14 µM) in 20 mM Tris-HCl buffer pH 8.5 at 25 °C; (x). rRNase II (1.8 µM) in 20 mM Tris-HCl buffer pH 8.5 at 37 °C; (xi). rRNase II (1.8 µM) in 20 mM Tris-HCl buffer pH 8.5 at 25 °C; (xii). rRNase II (14 µM) in 20 mM Tris-HCl buffer pH 8.5, Cu^2+^ 0.3 µM at 25 °C; (xiii). rRNase II (14 µM) in 20 mM Tris-HCl buffer pH 8.5, Cu^2+^ 1 µM at 25 °C; (xiv). rRNase III (14 µM) in 20 mM Tris-HCl buffer pH 8.5 at 25 °C; (xv). rRNase III (14 µM) in 20 mM Tris-HCl buffer pH 8.5 at 37 °C. At different times, the number of -SH/mole were determined using DTNB as titrant, and aliquots were taken to perform CD, fluorescence and activities analyses. 

Finally, (xvi). the oxidation of rRNase II using the mixture GSH/GSSG was performed according to the following procedure: rRNase II (2 µM) was incubated with GSH (2 mM) and GSSG (0.4 mM) in 50 mM sodium phosphate buffer pH 7.5, 5 mM EDTA at 30 °C. At different times an aliquot was analyzed for enzymatic activity.

### 4.4. RNase Activity Assay

Activities of native RNase, native RNase at different urea concentrations, rRNase I and II, rRNase III with different amount of disulfide bonds, rRNase IV with 1 mM EDTA, 0.2 mM DTT and 0.05 M urea, and re-oxidized rRNase (i-xvi as reported above) were assayed by the ribonuclease A detection kit (Sigma-Aldrich, St. Louis, MO, USA) which uses RNA as substrate [39]. The substrate RNA was essentially in a single strand conformation (double strand RNA ≈ 3%) as assayed spectrophotometrically after thermal denaturation. When necessary, the protein was diluted to a final concentration of 2.0 µM, in 50 mM sodium phosphate buffer pH 7.5, 5 mM EDTA. Only for the activity of the native protein at different urea concentrations, the protein was diluted to a final concentration of 2.0 µM, in 50 mM sodium acetate buffer pH 5.0 with different amounts of urea. An aliquot (RNase final concentration 80 nM) was then added in cuvette to a solution containing RNA (0.05% *w*/*v*), and 0.2 mM EDTA in 50 mM sodium acetate buffer pH 5.0. Only for rRNase IV, the concentrations of EDTA, urea and DTT were 40 µM, 2 mM and 8 µM, respectively. For the measurements of native RNase in 6 M, 7 M, and 8 M urea, the RNA was dissolved in the reaction buffer with 6 M, 7 M, or 8 M urea, respectively. The recovered activity was monitored spectrophotometrically in continuous at 300 nm, 25 °C. The data were reported as percentage of the ratio between sample and native RNase. Only in the case of native RNase at different urea concentrations, data were reported as normalized absorbance ratio between sample and native RNase.

### 4.5. Circular Dichroism Spectroscopy

CD spectra of native RNase, native RNase in 0.03 M urea, rRNase I, rRNase II, rRNase III, and rRNase IV in 0.03 M urea, 625 µM EDTA, 0.125 mM DTT were performed in 10 mM sodium phosphate buffer pH 7.4, at 25 °C, and in all cases with a protein concentration of 1.25 µM using a spectropolarimeter Jasco J-1500 (Easton, MD, USA). The setting panel was: slit 2 nm, sensibility 20 mdeg, resolution 0.5 nm, and range values 190–260 nm using a quartz cuvette of 0.5 cm light path. CD spectra of native RNase and rRNase in the presence of 0.03 M urea cannot be extended below 200 nm due to the interference of urea. CD spectra for all the samples of re-oxidized rRNase I and re-oxidized rRNase II were acquired in the same manner. The analyses of CD spectra were performed using DichroWeb [40] and BeStSel software [41].

### 4.6. Fluorescence Measurements

All the fluorescence analyses of native, reduced RNases (I–III) and re-oxidized rRNases (I and II) were performed on a Fluoromax-4 Horiba spectrofluorometer with an asymmetric quartz cuvette of 1 × 0.4 cm path length at 25 °C.

The fluorescence emission spectra of native, reduced RNases (I–III) and re-oxidized rRNases (I and II), in all cases 1.25 µM, were recorded in 10 mM sodium phosphate buffer pH 7.4 between 290 and 350 nm, using the following parameters: excitation wavelength of 275 nm and slits 5–8 nm.

### 4.7. Preparation of rRNase Samples for Mass-Spectrometry Analysis

The rRNase III at three different times of reduction (with a content of 2, 4, and 6 -SH/mole rRNase), was diluted to a final concentration of 1.25 μM in 10 mM sodium phosphate buffer with 1 mM bromopyruvic acid that alkylates residual protein cysteines within 1–2 s. Then, the samples were lyophilized. The samples (50 μg each one) were submitted to an overnight digestion at 3 °C using a porcine trypsin gold (mass spectrometry grade, resistant to autolytic digestion) in 1:50 (*w*/*w*) ratio with respect to the protein content. Enzymatic digestion was stopped by addition of 1 μL of 100% FA, and then 1μg of total protein content per sample was used for MS analyses.

### 4.8. HPLC-ESI-MS/MS Analysis

Nano-HPLC/nano-ESI-Orbitrap Elite analyses were performed on an UltiMate 3000 RSLCnano System coupled to an Orbitrap Elite MS detector with an EASY-Spray nano-ESI source (Thermo Fisher Scientific, Waltham, MA, USA). EASY-Spray column 15 cm × 50 μm ID, PepMap C18 (2 μm particles, 100 Å pore size) (Thermo Fisher Scientific), was used for bottom-up analyses, in coupling to an Acclaim PepMap 100 cartridge (C18, 5 μm, 100 Å, 300 μm i.d. × 5 mm) (Thermo Fisher Scientific). Bottom-up nano-HPLC-MS/MS analyses were performed using aqueous solution of FA (0.1%, *v*/*v*) as eluent A and ACN/FA (99.9:0.1, *v*/*v*) as eluent B in the following gradient elution: (a) 5% B (2 min), (b) from 5% to 60% B (30 min), (c) from 60% B to 99% (10 min), (d) 99% B (10 min), (e) from 99% to 5% B (2 min), and (f) 5% B (10 min) at a flow rate of 0.3 μL/min. The injection volume was 5 μL corresponding to 1 μg of total protein content per sample. Peptide trapping and concentration were obtained by loading the sample for 5 min into the Acclaim PepMap 100 nano-trap cartridge operating at 10 μL/min in eluent A. Chromatographic separations were performed at 35 °C. The Orbitrap Elite instrument was operating in positive ionization mode, performing MS/MS fragmentation by higher energy collisional dissociation (HCD) of the five most intense signals of each spectrum, measured at a 60,000 resolution in 150–2000 *m*/*z* acquisition range, in data-dependent scan (DDS) mode. The minimum signal was set to 500.0, the isolation width to 2 *m*/*z*, the default charge state to +2, and the activation Q to 0.25 MS/MS spectra acquisition was performed in the Orbitrap at 60,000 resolution.

### 4.9. Data and Graphical Representation

The experimental data reported in Figures and Tables were analyzed and expressed as Mean ± Standard Deviation (S.D.). Data were obtained from three independent experiments performed in different days by the same operators using the same instruments. The graphic and results visualization were obtained using GraphPad Prism software v5.0 (La Jolla, CA, USA). Crystal structure of native RNase is derived by PDB id: 1FS3 [42], the structure was drawn by UCSF Chimera [43].

## Figures and Tables

**Table 1:** Experiments of re-oxidation of rRNases.

Sample	T	[rRNase] (µM)	[Cu^2+^] (µM)	t (h)	Activity (%)
rRNase I	37 °C	14	-	49	23
	25 °C	14	-	49.6	47
	37 °C	9.2	10	1	7
	25 °C	14	0.3	8.3	41
	25 °C ^a^	14	0.3	19.7	82
	25 °C	14	1	2.2	27
	25 °C	14	10	1	9
rRNase II	37 °C	14	-	106	11
	25 °C	14	-	106	49
	37 °C	1.8	-	92	2.5
	25 °C	1.8	-	92	23
	25 °C	14	0.3	7	37
	25 °C	14	1	5.5	25
rRNase III	37 °C	14	-	46	25
	25 °C	14	-	50	35

^a^ With β-ME 11 µM.

**Table 2:** Secondary structure analysis of the CD spectra of re-oxidized rRNase I.

Sample	T	[Cu^2+^] (µM)	Helix	Strand	Turn	Other
Native	-	-	14.2%	27.5%	20.6%	37.7%
Re-oxidized rRNase I	37 °C	-	12.2%	22.5%	18.0%	47.3%
	37 °C	10	10.2%	19.1%	16.3%	54.4%
	25 °C	0.3	15.0%	21.3%	19.0%	44.7%
	25 °C	1	13.7%	19.0%	17.3%	50.0%
	25 °C	10	17.3%	9.7%	14.8%	58.1%

**Table 3:** Secondary structure analysis of the CD spectra of re-oxidized rRNase II.

Sample	T	[Cu^2+^] (µM)	Helix	Strand	Turn	Other
Native	-	-	14.2%	27.5%	20.6%	37.7%
Re-oxidized rRNase II	37 °C	-	10.4%	22.2%	17.5%	49.9%
	25 °C	-	15.5%	21.0%	19.2%	44.2%
	25 °C	0.3	21.0%	24.7%	19.4%	34.9%
	25 °C	1	28.6%	13.5%	16.6%	41.3%

**Table 4:** Secondary structure analysis of the CD spectra of rRNases.

Sample	Urea	G-25	Helix	Strand	Turn	Other
Native	-	-	14.2%	27.5%	20.6%	37.7%
rRNase I	Yes	pH 7.4	9.7%	10.0%	13.1%	67.2%
rRNase II	Yes	Acetic Acid	8.4%	16.0%	14.1%	61.4%
rRNase III	No	pH 7.4	9.2%	14.5%	14.1%	62.3%

**Table 5:** Secondary structure analysis of the CD spectra of the reduction pathway.

Sample	-SH/mole	Helix	Strand	Turn	Other
Native	-	14.2%	27.5%	20.6%	37.7%
Partially rRNase	1.9	15.5%	21.2%	19.2%	44.4%
	4.2	14.9%	16.0%	17.2%	51.9%
	6.0	15.5%	10.0%	14.7%	59.9%
rRNase III	7.5	9.2%	14.5%	14.1%	62.3%

**Table 6:** Secondary structure analysis of the CD spectra of rRNase IV.

Sample	[Urea] (M)	Incubation Time	Helix	Strand	Turn	Other
Native	0.03	1 h	16.2%	32.5%	12.6%	38.7%
rRNase IV	0.03	1 h	4.3%	23.6%	16.7%	55.4%
rRNase IV	0.03	24 h	5.4%	23.5%	16.3%	54.8%

## Data Availability

Data are contained within the article.
